# Renal Amyloidosis: An Approach to Uniform Biopsy Reporting

**DOI:** 10.7759/cureus.113520

**Published:** 2026-07-28

**Authors:** Mohita Ray, Biswajit Mishra, Nibedita Sahoo, Biswaranjan Mohanty

**Affiliations:** 1 Department of Pathology, IMS &amp; SUM Hospital, Siksha 'O' Anusandhan (Deemed University), Bhubaneswar, IND; 2 Department of Nephrology, IMS &amp; SUM Hospital, Siksha 'O' Anusandhan (Deemed University), Bhubaneswar, IND

**Keywords:** amyloid typing, grading, histopathology, immunofluorescence, renal amyloidosis

## Abstract

Background: Renal amyloidosis results in progressive renal failure, and consistent biopsy reporting improves prognostic accuracy and permits interinstitutional comparisons.

Objectives: This study aims to apply the histopathologic classification of Sen and Sarsik, calculate the Renal Amyloid Prognostic Score (RAPS), and correlate histological grade with clinical severity.

Methods: We retrospectively analyzed 18 biopsy-proven cases of renal amyloidosis out of 1,008 native renal biopsies performed at IMS & SUM Hospital, Siksha 'O' Anusandhan (Deemed University), Bhubaneswar, Odisha, India, between January 2021 and January 2026. Glomerular amyloid pattern (GAP) classes I-VI, RAPS, and final histologic grade (0-3) were assigned. Clinical data included age, sex, 24-hour protein, urea, creatinine, and underlying diagnosis. Statistical analyses used independent t tests, Fisher’s exact test, and Spearman correlation.

Results: The prevalence was 1.78%. The majority were primary amyloid light chain (AL) (12/18). Glomerular deposition was universal, with GAP class IV (15/18) predominating, associated with Grade 3. Grade 3 had significantly higher 24-hour proteinuria and serum creatinine than Grade 2 (P = 0.006 and P = 0.042). RAPS correlated positively with proteinuria (r = 0.68, P < 0.01) and creatinine (r = 0.59, P < 0.05).

Conclusions: RAPS and histologic grading provide a consistent evaluation of renal amyloid burden, correlating with clinical severity. Prompt biopsies and standardized reporting can inform prognosis and guide treatment.

## Introduction

Amyloidosis is a group of disorders characterized by the extracellular deposition of insoluble fibrillar proteins, which exhibit distinctive staining properties, such as Congo red positivity with apple green birefringence under polarized light [1,2].

Although systemic amyloidosis can affect any organ, the kidney is the most frequently involved site. However, cardiac involvement remains the leading cause of mortality in systemic amyloidosis. The literature shows that primary amyloid light chain (AL) and secondary amyloid A (AA) amyloidosis are the most prevalent forms of amyloidosis in renal tissue [3,4]. Asian countries have a higher prevalence of secondary amyloidosis, despite Western countries considering primary amyloidosis to be the most frequent kind [5].

Amyloid deposits can be observed histologically as salmon-pink deposits on Congo red stain with apple-green birefringence when examined under a polarising microscope [6]. Immunofluorescence (IF) for kappa and lambda light chains and immunohistochemistry (IHC) for serum amyloid A (SAA) protein determine if renal amyloidosis is primary or secondary. When it comes to prognosis and treatment, knowing the subtype of renal amyloidosis is crucial [6]. Some of the symptoms of renal amyloidosis include proteinuria, nephrotic syndrome, hypertension, and renal failure [7].

Systemic amyloidosis affects the kidney frequently and is a leading cause of mortality (second only to cardiac involvement) regardless of the cause [8]. There is a wide range of renal morphological alterations, and the main location of involvement can be influenced by the chemical composition to some extent. However, the glomerulus is the principal location for the early production of fibrils [4,9]. Additional abnormalities that cause significant morbidity include glomerular sclerosis, interstitial inflammation, fibrosis, and tubular atrophy [7,10]. Renal amyloidosis histopathologic features have evolved alongside the identification and categorization of new lesions [10-20].

Based on the results of renal biopsy studies, there needs to be standardization in the reporting of renal amyloid biopsy results, which includes biochemical and histopathologic classifications, assessments of renal amyloid deposition, and association with other histopathologic lesions and grades. Critical for patient treatment, comparing results and therapeutic trials across clinics, and assessing disease extent and renal amyloid load, standardizing the interpretation of renal amyloid biopsies is essential.

The scoring and grading system proposed by Sen and Sarsik [9] offers an improved method of predicting results and evaluating treatment trials. They have also tried to standardize renal amyloidosis reporting to allow for interinstitutional comparability and consistency in renal amyloid biopsy reporting.

The primary objective of the study was to evaluate the prognostic value of standardized histopathological grading (Sen and Sarsik classification and Renal Amyloid Prognostic Score (RAPS)) in renal amyloidosis and its correlation with clinical severity.

The secondary objectives were to describe the clinicopathological spectrum of renal amyloidosis in a tertiary care cohort, to compare histological grades between primary (AL) and secondary (AA) amyloidosis, and to assess the relationship between histological grade and renal function parameters (proteinuria, serum creatinine, and urea).

Research hypothesis

We hypothesize that higher histological grades of renal amyloidosis, as defined by the Sen and Sarsik classification and RAPS, are significantly associated with greater clinical severity (increased proteinuria and renal dysfunction).

## Materials and methods

From January 2021 through January 2026, all cases of renal amyloidosis that were confirmed by biopsy were included in this record-based analysis. This retrospective study was conducted in the Department of Pathology, Institute of Medical Sciences (IMS) and Sum Hospital, Bhubaneswar, Odisha, India. Clinical data were collected from medical records.

Inclusion criteria were a minimum of 10 glomeruli for light microscopy (LM), a minimum of one glomerulus for IF, and all cases with a histopathological diagnosis of renal amyloidosis. Transplant biopsies were not included.

Urine protein (24 hours) (>3.5 g = nephrotic range), serum urea (normal range 6-24 mg/dL), serum creatinine, and serum protein electrophoresis were among the laboratory values that were retrieved. History of hypertension, chronic inflammatory disorders, chronic kidney disease, renal failure, and plasma cell dyscrasias were collected from the patient's medical history.

Two kidney biopsy cores were sent for pathological analysis using IF in phosphate-buffered saline and light microscopy in 10% buffered formalin. Frozen renal biopsies were subjected to direct IF with antibodies specific to Kappa, Lambda, IgG, IgA, IgM, C3, and C1q coupled to fluorescein isothiocyanate (FITC). An Olympus BX43 equipped with a FITC filter was used for the analysis of the slides.

For histopathological examination, 2-4 µm sections were used for hematoxylin and eosin (H&E), periodic acid-Schiff (PAS), Masson's trichrome (MT), and Jones methenamine silver (JMS). Then, 8-10 µm thick sections were used for Congo red staining.

All biopsies were independently evaluated by two nephropathologists (MR, NS), and a consensus opinion was used for scoring and final diagnosis in cooperation with the nephrologists.

The presence of amyloid deposits in the glomerular, interstitial, vascular, or all compartments was determined by analyzing the biopsies. The extracellular amyloid material appeared amorphous and eosinophilic on H&E, pale to negative on PAS, negative on silver staining, and gray-blue on MT. Under polarized light, it exhibited apple-green birefringence and tested positive with Congo red. We looked at whether the main site of involvement was in the glomeruli, the interstitial spaces, the blood vessels, or in every compartment. Based on the pattern of involvement, Sen and Sarsik classified glomerular involvement into six classes, referred to as the glomerular amyloid pattern (GAP). GAP class, glomerular, interstitial, and vascular compartment involvement, as well as interstitial fibrosis, tubular atrophy, glomerular sclerosis and inflammation, were all factors in amyloid scoring. The RAPS was the sum of all the scores. The RAPS provided the final histological grade from 0 to 3.

Renal impairment was defined by elevated serum creatinine (>1.3 mg/dL), elevated blood urea (>40 mg/dL), and presence of chronic kidney disease or acute kidney injury as documented in patient records. Clinical severity was defined by nephrotic range proteinuria (>3.5 g/24 h), presence of hypertension, and progression to renal failure. These parameters will be correlated with histological grade and RAPS scores.

There are two types of amyloidosis: 1) primary amyloidosis (light-chain restriction shown by direct IF and supplementary data) in plasma cell monoclonal proliferation and 2) secondary amyloidosis (no light-chain restriction, chronic inflammatory background). No further subtype classification of secondary amyloidosis was performed due to the absence of immunohistochemistry for serum amyloid A and relevant clinical data.

## Results

We report on a five-year study of renal amyloidosis in 18 cases picked from 1,008 renal samples sent to the pathology department of IMS and Sum Hospital, Bhubaneswar, from January 2021 to January 2026. The prevalence was 1.78% of all the renal biopsies received in the study period. The 18 patients ranged in age from 32 to 69 years, with a mean of 53.16 years. There were 12 males and six females, for a male-to-female ratio of 2:1. The average creatinine level was 1.49 mg/dl, and the average proteinuria was 3.8 g/24 hours.

The presenting manifestation was nephrotic syndrome. Of these, four patients had concomitant hypertension, and two cases had acute kidney injury in addition to chronic kidney disease. Concomitant anaemia was present in two of the 10 cases; one of these showed a positive M-band on serum protein electrophoresis. One case was a diagnosed case of non-Hodgkin’s lymphoma on therapy. One case was a diagnosed case of rheumatoid arthritis.

**Figure 1:**
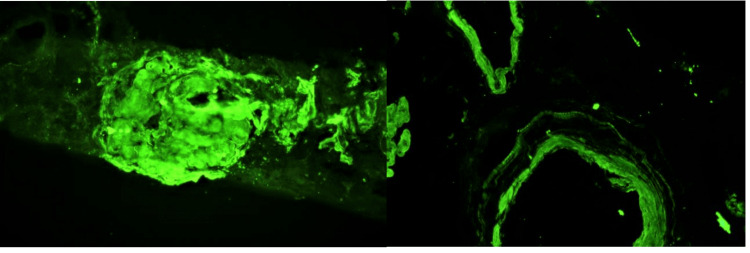
A case of AL amyloidosis showing Lambda positivity in glomeruli (A) and blood vessel walls (B)

Eighteen cases were identified, of which 12 cases were classified as primary amyloidosis (light chain restriction by direct IF), and the remaining six cases were classified as secondary amyloidosis. Four of the 12 patients with primary amyloidosis were subsequently found to have multiple myeloma. All others were diagnosed as monoclonal gammopathy of renal significance (MGRS). Eleven of these were restricted to lambda light chains and one to kappa light chains (Figure 1).

**Figure 2:**
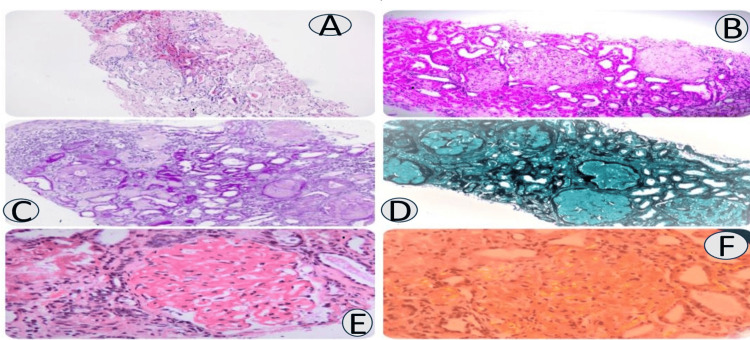
A case of GAP Class IV: A. Glomerular mesangiocapillary and interstitial amyloid deposits. B. PAS-negative glomerular deposits. C. PAS-negative glomerular and interstitial deposists. D. Silver negative glomerular deposits. E. Deposits are salmon pink on Congo-red. F. Congo red shows apple-green birefringence on polarizer.

Histopathological analysis revealed that, in the present study, renal involvement was predominantly glomerular (100%) with involvement of other compartments as well. There was no exclusive involvement of glomeruli, interstitium, or vasculature. No differences were observed in the distribution of amyloid across various renal compartments in primary and secondary amyloidosis cases. Histological findings show significant glomerular involvement in all groups. Figure 2 depicts the majority of glomerular amyloid deposits classified as GAP Class IV.

Following the final assessment of the biopsy results, most cases were graded as Grade 3. This was strongly associated with GAP Class IV. Table 1 shows the baseline demographics and clinical features of primary versus secondary renal amyloidosis. Table 2 depicts the distribution of histological biopsy grades by amyloid type.

**Table 1:** Baseline demographics and clinical characteristics (primary vs. secondary)

Parameter	Primary / AL (n = 12) Mean ± SD	Secondary / AA (n = 6) Mean ± SD	Total cohort (n = 18) Mean ± SD
Age (years)	57.00 ± 10.85	45.50 ± 13.19	53.17 ± 12.59
Sex (male:female)	9:3	3:3	12:6
24-hour protein (g/day)	4.81 ± 2.71	5.00 ± 1.45	4.87 ± 2.32
Blood urea (mg/dL)	41.49 ± 20.72	30.73 ± 12.88	37.91 ± 18.81
Serum creatinine (mg/dL)	1.50 ± 0.38	1.46 ± 0.69	1.49 ± 0.48

**Table 2:** Distribution of histological biopsy grades by amyloid type

Histological grade	Primary / AL (n = 12) (%)	Secondary / AA (n = 6) (%)	Total (n = 18)
Grade I	0 (0%)	0 (0%)	0
Grade II	2 (17%)	2 (33%)	4
Grade III	10 (83%)	4 (67%)	14

Table 3 summarizes the group comparison of clinical parameters between histologic grades 2 and 3, showing the correlation of the parameters with the histologic grade.

**Table 3:** Clinical parameters by histologic grade Independent t‑test; P < 0.05 considered statistically significant.

Parameter	Grade II (Mean ± SD)	Grade III (Mean ± SD)	P value	Significance
Age (years)	43.7 ± 13.9	59.6 ± 9.1	0.060	NS
24-hour urine protein (gm/24 hours)	3.77 ± 0.25	5.71 ± 0.86	0.006	Significant
Urea (mg/dl)	21.0 ± 4.0	49.8 ± 22.0	0.061	NS
Creatinine (mg/dl)	0.92 ± 0.33	1.58 ± 0.41	0.042	Significant

Significant differences were noted in 24-hour urine protein (p = 0.006) and serum creatinine (p = 0.042), which indicate that with worsening histologic grade, proteinuria and renal dysfunction also progress.

## Discussion

**Figure 3:**
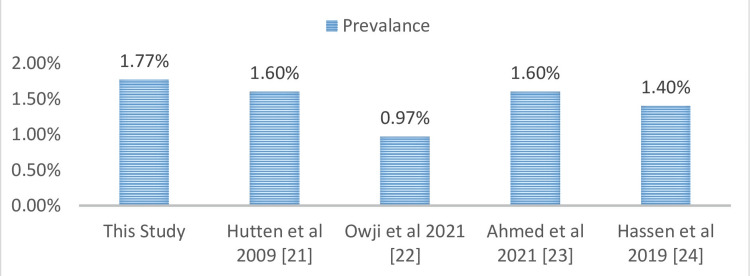
Prevalence of renal amyloidosis

Our 18 reported cases of renal amyloidosis span the course of the five years of the study, accounting for approximately 1.78% of all renal biopsies performed during that time. As indicated in Graph 1 (Figure 3), this prevalence was similar to that found in studies by Hutten et al., Owji et al., Ahmed et al., and Hassen et al., which were 1.60%, 0.97%, 1.60%, and 1.40%, respectively [21-24].

**Figure 4:**
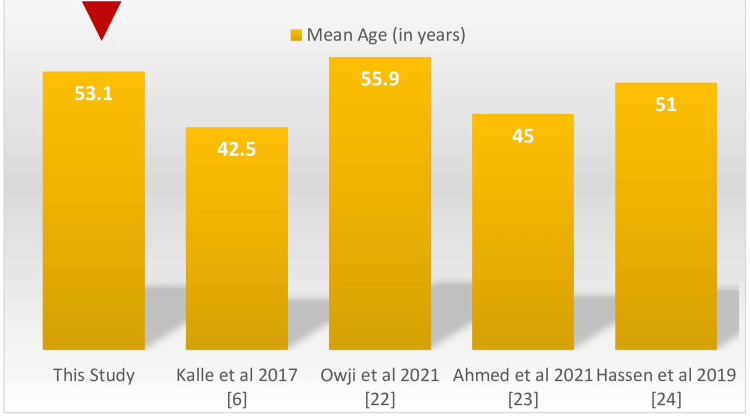
Graph of the mean age distribution of the study

Patients included in our study had a mean age of 53.16 years. Graph 2 (Figure 4) demonstrates that the average age of the participants was comparable to previous studies conducted by Ahmed et al., Kalle et al., Owji et al., and Hassen et al. [22-24].

**Figure 5:**
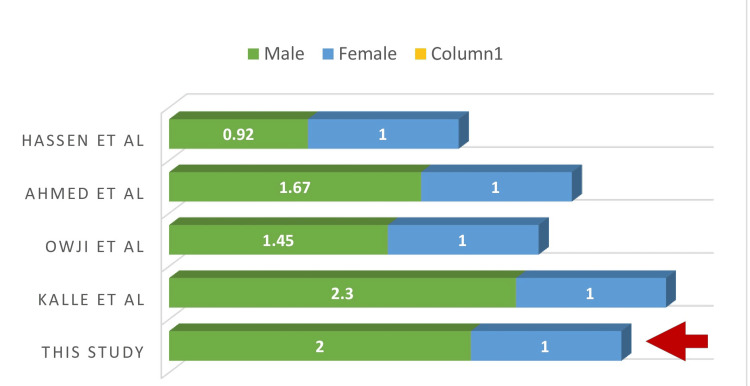
Graph showing male and female renal amyloidosis ratio of the study

According to the study conducted by Kalle et al., the mean age of patients with AL amyloidosis was 57 years, which is greater than the mean age of patients with AA amyloidosis [6]. These data point to the elderly as the most common age group affected by AL amyloidosis. Graph 3 (Figure 5) shows that there were twice as many men as women in our sample, which is in line with previous research that has demonstrated a male majority in cases of renal amyloidosis [22-24].

This cohort demonstrates the clinicopathological spectrum of renal amyloidosis, with clear differences between AL and AA. Epidemiological data indicate AL predominance in older populations with plasma cell dyscrasias and AA association with chronic inflammatory conditions in younger people [25-28]. Patients with AL amyloidosis were older (mean 57 years) than AA (45.5 years). Both groups had high proteinuria (~5 g/day), which is consistent with the glomerular involvement typical of renal amyloidosis.

Histological severity correlated well with renal impairment. Biopsies of Grade 3 (83% of AL, 67% of AA) had increased serum creatinine (1.64 vs. 0.94 mg/dL) and increased RAPS scores (18.7 vs. 13.7), emphasizing the prognostic value of histologic grading. Recent investigations have confirmed that glomerular amyloid burden and vascular involvement are independent predictors of renal progression, with higher grades associated with rapid deterioration in renal survival [26,29].

Prognostic grading was applied to outcomes as well. Furthermore, 83% of patients were in GAP class 4 with diffuse mesangiocapillary deposition. GAP and RAPS scoring have been validated as robust predictors for renal survival in contemporary research, emphasizing their value in clinical practice [26,30]. Interstitial fibrosis was also associated with renal function in a stepwise fashion, with creatinine levels of 1.11 mg/dL for score 1 and 1.80 mg/dL for score 4. In addition to glomerular scoring systems [29], tubulointerstitial fibrosis is now being recognised as a late marker of irreversible renal decline.

Glomerulosclerosis was rare (11% moderate sclerosis), but even mild sclerosis accelerates progression of chronic renal disease. This finding is consistent with the larger literature on nephrotic syndrome, where sclerosis is a marker of secondary glomerular injury and is associated with worse prognosis [29].

Treatment is variable, so early biopsy and amyloid typing are still important: chemotherapy (eg, daratumumab-CyBorD) for AL and anti-inflammatory treatment for AA. New anti-fibril immunotherapies and antifibrotic medicine in testing in CKD may improve results even further. Histological scoring together with new medicines may allow for customized therapy of renal amyloidosis [26,28,30].

A major limitation of this study was the absence of SAA immunohistochemistry, which restricted definitive typing of secondary (AA) amyloidosis. In our cohort, AA cases were inferred indirectly, based on the absence of light chain restriction and the presence of chronic inflammatory conditions. While this pragmatic approach allowed classification, it introduces diagnostic uncertainty and raises the possibility of misclassification. Consequently, conclusions regarding differences between AL and AA groups must be interpreted with caution. Future studies should incorporate IHC or mass spectrometry to ensure accurate amyloid typing, as precise classification is critical for both prognostic assessment and therapeutic decision-making.

## Conclusions

The prevalence, mean age of presentation, M:F ratio, and the most prevalent clinical manifestation of nephrotic syndrome (due to predominance of glomerular involvement) in our study were comparable to other studies globally. Male and older patients were associated with higher histological grade.

We tried to grade and score amyloid according to the recommendations by Sen S et al. Most of our cases were Grade 4, and we found a good association between GAP Class 4 and Grade 3. Histologic grade showed a statistically significant association with degree of proteinuria (24 hours) and creatinine. We conclude that RAPS and histologic grading as per Sen and Sarsik can give an overall picture of the disease load in renal amyloidosis.

In our study, we found that the majority of the cases were in GAP Class 4, followed by Class 6, with nephrotic range proteinuria. Early biopsy can detect disease in lower GAP Class, where early medical intervention can result in better patient prognosis.

The most frequent diagnosis was AL amyloidosis, which is comparable to the studies from the Western world. This may be possible due to low socioeconomic status and an uninformed attitude of people to healthcare, resulting in chronic diseases remaining undetected.

Due to the close correlation between histological scoring and clinical symptoms, future studies might focus on follow-up renal biopsies after completing therapy to evaluate histological remission as a surrogate marker for definitive treatment response and disease prognosis.

Furthermore, long-term multi-institutional studies with follow-up can help in understanding the biological behaviour of the disease.
